# Supplementary material for: Environmental Correlates of Sexual Signaling in the Heteroptera: A Prospective Study
Source: Insects. 2021 Nov 30;12(12):1079. doi: 10.3390/insects12121079 (PMC8707444; doi:10.3390/insects12121079)
Supplement: Supplementary file 1 [file insects-12-01079-s001.zip › insects-1433493-supplementary.pdf]

**Supplementary material**

**Table S1:** A table of all articles used in the collection of data about Heteropteran families. Four modes of sexual signals (stridulation, abdominal vibration, chemical signals and antennation) and three habitat types (plants, semi-aquatic and leaf litter / ground dwelling) were chosen to be included within the analyses. The sexual signal modes and habitats of each family were researched thoroughly and scored as present (1) or absent (0) based on the chosen signals and habitats. If a family exhibited more than one form of sexual signal or was present in more than one habitat they were marked as present in both. If a family had no available information or had a mode of signalling or habitat type that did not fit into the categories chosen they were marked as absent. The concluding data was analysed using binomial regression analyses to see if signalling mode could be predicted by habitat type. See reference list for full reference.

| **Family** | **Common names** | **References** |
|---|---|---|
| Acanthosomatidae | | [1–4] |
| Aenictopecheidae | | NA |
| Aepophilidae | | [5] |
| Alydidae | Broad-headed bugs | [6–12] |
| Anthocoridae | Flower bugs / minute pirate bugs | [13,14] |
| Aphelocheiridae | | [15,16] |
| Aradidae | Flat bugs | [2,17,18] |
| Belostomatidae | Giant water bugs | [18–22] |
| Berytidae | Stilt bugs | [23–26] |
| Canopidae | | [5] |
| Ceratocombidae | | [17] |

| | | |
|---|---|---|
| Cimicidae | Bed bugs / bat bugs / bird bugs | [27–29] |
| Colobathristidae | | [2] |
| Coreidae | Leaf-footed bugs / squash bugs | [2,18,30–32] |
| Corimelaenidae | | [33,34] |
| Corixidae | Water boatmen | [35–38] |
| Cydnidae | Burrower bugs | [17,39–41] |
| Dipsocoridae | Jumping around bugs | [25,42] |
| Enicocephalidae | Unique-headed bugs / gnat bugs | [43,44] |
| Gelastocoridae | Toad bugs | [5,43,45] |
| Gerridae | Water-striders / pond skaters | [18,46–49] |
| Hebridae | Velvet water bugs | [5,15,46,50] |
| Helotrephidae | Beetle back swimmers | [5,15,16,51,52] |
| Hermatobatidae | | [46,47] |
| Hydrometridae | Water measurers | [5,16,46] |
| Hyocephalidae | | [25,53–55] |
| Hypsipterygidae | | [56–58] |
| Idiostolidae | | [59–61] |

| | | |
|---|---|---|
| Joppeicidae | | [5,62,63] |
| Largidae | | [2,64–68] |
| Lasiochilidae | Flower bugs | [69–71] |
| Leptopodidae | Spiny shore bugs | [5,15,16] |
| Lestoniidae | | [72–74] |
| Lyctocoridae | | [14,69,70,75,76] |
| Lygaeidae | Seed bugs | [56,77–83] |
| Macroveliidae | Macroveliid shore bugs | [15] |
| Malcidae | | [5,84,85] |
| Medocostidae | | [58,86] |
| Megarididae | | [87,88] |
| Mesoveliidae | Water treaders | [15,16,46] |
| Microphysidae | | [5] |
| Miridae | Leaf bugs / Plant bugs | [14,87,89–91] |
| Nabidae | Damsel bugs | [87,89,92] |
| Naucoridae | Creeping water bug | [15,16,93,94] |
| Nepidae | Water scorpions | [15,51,95–97] |
| Notonectidae | Backswimmers | [15,51,98,99] |
| Ochteridae | Velvety shore bug | [15,52] |
| Omaniidae | | [15,100] |

| | | |
|---|---|---|
| Pachynomidae | | [5,17] |
| Paraphrynoveliidae | | [15] |
| Parastrachiinae | | [5,101,102] |
| Pentatomidae | Stink bugs / solider bugs | [2,5,103–105] |
| Phloeidae | | [5,77,106–108] |
| Piesmatidae | Ash-grey leaf bugs | [5,109–111] |
| Plataspidae | | [2,112–114] |
| Pleidae | Pygmy backswimmers | [5,25,36,51,115] |
| Plokiophilidae | | [5,14,116] |
| Polyctenidae | Bat bugs | [5,117] |
| Potamocoridae | | [15,52,118] |
| Pyrrhocoridea | | [5,25,67,119–121] |
| Reduviidae | Assassin bugs / ambush bugs / thread-legged bugs | [5,18,25,122–125] |
| Rhopalidae | Scentless plant bug | [5,25,126–128] |
| Saileriolidae | | NA |
| Saldidae | Shore bugs | [5,15,129] |
| Schizopteridae | | [56,130] |
| Scutelleridae | Shield-back bugs | [43,131] |

| | | |
|---|---|---|
| Stemmocryptidae | | [50,56,57,132] |
| Stenocephalidae | | [5,133] |
| Termitaphididae | | [5,134,135] |
| Tessaratomidae | | [2,5,87,110,136] |
| Thaumastellidae | | [2,5,137,138] |
| Thaumastocoridae | Royal palm bugs | [5,139–141] |
| Tingidae | Lace bugs | [5,25,87,142] |
| Urostylididae | | [143,144] |
| Veliidae | Broad-shouldered water striders | [46] |
| Velocipedidae | | [145,146] |
| **All families included data from Panizzi and Grazia (2015)** [147] | | |

## References

1. Carvajal, M.A.; Faúndez, E.I. Revalidation of Ditomotarsus hyadesi Signoret, 1885 stat. rest.(Hemiptera: Heteroptera: Acanthosomatidae) with notes on its Natural History. *Biodivers. Nat. Hist.* **2015**, *1*, 18–25.

2. Čokl, A. Stink bug interaction with host plants during communication. *J. Insect Physiol.* **2008**, *54*, 1113–1124.

3. Gogala, M. Sound or Vibration, an Old Question of Insect Communication. In *Studying Vibrational Communication. Animal Signals and Communication, vol 3. Springer: New York, USA.*; 2014; pp. 31–46.

4. Kaitala, A.; Mappes, J. Parental care and reproductive investment in shield bugs (Acanthosomatidae, Heteroptera). *Oikos* **1997**, 3–7.

5. Schaefer, C.W. Prosorrhyncha: Heteroptera and Coleorrhyncha. In *Encyclopedia of insects. Academic Press: Cambridge, Massachusetts, USA.*; 2009; pp. 839–855.

6. Sakurai, T. Multiple Mating and Its Effect on Female Reproductive Output in the Bean Bug Reptortus clavatus (Heteroptera: Alydidae). *Ann. Entomol. Soc. Am.* **1996**, *89*, 481–485, doi:10.1093/aesa/89.3.481.

7. Aldrich, J.R.; Zhang, A.; Oliver, J.E. Attractant pheromone and allomone from the metathoracic scent gland of a broad-headed bug (Hemiptera: Alydidae). *Can. Entomol.* **2000**, *132*, 915–923.

8. Ishizaki, M.; Watanabe, T.; Moriya, S.; Tabuchi, K. Diurnal locomotion activity of adult rice bug, Leptocorisa chinensis (Hemiptera: Alydidae), at different ages, measured by actograph and video camera. *Appl. Entomol. Zool.* **2011**, *46*, 135–142.

9. Numata, H.; Kon, M.; Fujii, H.; Hidaka, T. Sound production in the bean bug, Riptortus clavatus Thunberg (Heteroptera: Alydidae). *Appl. Entomol. Zool.* **1989**, *24*, 169–173.

10. Jung, J.K.; Im, D.J. Attraction of the bean bug, Riptortus clavatus (Thunberg)(Hemiptera: Alydidae), by opposite sexes in a soybean field. *J. Asia. Pac. Entomol.* **2003**, *6*, 239–241.

11. Mizutani, N.; Wada, T.; Yasuda, T.; Endo, N.; Yamaguchi, T.; Moriya, S. Influence of photoperiod on attractiveness and pheromone contents of the bean bug, Riptortus pedestris (Heteroptera: Alydidae). *Appl. Entomol. Zool.* **2008**, *43*, 585–592.

12. Suzaki, Y.; Katsuki, M.; Miyatake, T.; Okada, Y. Male courtship behavior and weapon trait as indicators of indirect benefit in the bean bug, Riptortus pedestris. *PLoS One* **2013**, *8*, e83278, doi:10.1371/journal.pone.0083278.

13. Peet, W.B. Description and Biology of Nidicola jaegeri, n. sp., from Southern California (Hemiptera: Anthocoridae). *Ann. Entomol. Soc. Am.* **1979**, *72*, 430–437.

14. Tatarnic, N.J.; Cassis, G.; Hochuli, D.F. Traumatic insemination in the plant bug genus Coridromius Signoret (Heteroptera: Miridae). *Biol. Lett.* **2005**, *2*, 58–61.

15. Lytle, D.A. Order Hemiptera. In *Thorp and Covich's freshwater invertebrates. Academic Press: Cambridge, Massachusetts, USA.*; 2015; pp. 951–963.

16. Yang, C.M.; Kovac, D.; Cheng, L. Insecta: Hemiptera, Heteroptera. In *Freshwater Invertebrates of the Malaysian Region. Academy of Sciences Malaysia. UC San Diego: Marine Biology Research Division.*; 2004; pp. 457–490.

17. Pluot-Sigwalt, D. A pair of basi-abdominal sex pheromone glands in the male of some burrower bugs (Hemiptera: Heteroptera: Cydnidae). *Acta Entomol. musei Natl. pragae* **2008**, *48*, 511–522.

18. Smith, R.L. Evolution of exclusive postcopulatory paternal care in the insects. *Florida Entomol.* **1980**, *63*, 65–78.

19. Kraus, W.F. Surface wave communication during courtship in the giant water bug, Abedus indentatus (Heteroptera: Belostomatidae). *J. Kansas Entomol. Soc.* **1989**, 316–328.

20. Smith, R.L. Paternity assurance and altered roles in the mating behaviour of a giant water bug, Abedus herberti (heteroptera: Belostomatidae). *Anim. Behav.* **1979**, *27*, 716-725.

21. Smith, R.L. Male Brooding Behavior of the Water Bug Abedus herberti (Hemiptera: Belostomatidae). *Ann. Entomol. Soc. Am.* **1976**, *69*, 740–747.

22. Ichikawa, N. Egg mass destroying behaviour of the female giant water bug Lethocerus deyrollei Vuillefroy (Heteroptera: Belostomatidae). *J. Ethol.* **1990**, *8*, 5–11, doi:10.1007/BF02350123.

23. Wheeler, A.G. Neides muticus (Hemiptera: Berytidae): life History and Description of Fifth Instar. *Ann. Entomol. Soc. Am.* **1978**, *71*, 733–736.

24. Costas, M.; Vázquez, Á. Stilt Bugs (Hemiptera: Berytidae). In *Encyclopedia of Entomology. Springer: Boston, USA.*; 2008; pp. 3566–3567.

25. Aldrich, J.R. Chemical ecology of the Heteroptera. *Annu. Rev. Entomol.* **1988**, *33*, 211–238.

26. Howe, M.A. A provisional checklist of the invertebrates recorded from Wales. *Brachyceran flies (Diptera Xylophagidae to Dolichopodidae)* **2002**, *3*, 1–84.

27. Usinger, R.L. *Monograph of Cimicidae (Hemiptera, Heteroptera). Entomological society of America.*; 1966;

28. Miller, D. Bed Bugs (Hemiptera: Cimicidae: Cimex spp.). Springer: Boston, USA. In *Encyclopedia of Entomology*; 2008; pp. 405–417.

29. Reinhardt, K.; Siva-Jothy, M.T. Biology of the bed bugs (Cimicidae). *Annu. Rev. Entomol.* **2007**, *52*, 351–374.

30. Eberhard, W.G. Sexual Behavior of Acanthocephala declivis guatemalana (Hemiptera: Coreidae) and the Allometric Scaling of their Modified Hind Legs. *Ann. Entomol. Soc. Am.* **1998**, *91*, 863–871.

31. McCullough, T. Acid content of scent fluid from Acanthocephala femorata, A. declivis, and A. granulosa (Hemiptera: Coreidae). *Ann. Entomol. Soc. Am.* **1970**, *63*, 1199–1199.

32. Wang, Q.; Millar, J.G. Mating behavior and evidence for male-produced sex pheromones in Leptoglossus clypealis (Heteroptera: Coreidae). *Ann. Entomol. Soc. Am.* **2000**, *93*, 972–976.

33. Hoffman, R.L. Shield Bugs:(Hemiptera; Scutelleroidea; Scutelleridae, Corimelaenidae, Cydnidae, Pentatomidae). In *The insects of Virginia. Virginia Polytechnic Institute and State University.*; 1971.

34. Biehler, J.A.; Mcpherson, J.E. Life History and Laboratory Rearing of Galgupha ovalis (Hemiptera: Corimelaenidae), with Descriptions of Immature Stages1. *Ann. Entomol. Soc. Am.* **1982**, *75*, 465–470.

35. Aiken, R.B. Sound production and mating in a waterboatman, Palmacorixa nana (Heteroptera: Corixidae). *Anim. Behav.* **1982**, *30*, 54–61.

36. Aiken, R.B. Effects of group density on call rate, phonokinesis, and mating success in Palmacorixa nana (Heteroptera: Corixidae). *Can. J. Zool.* **1982**, *60*, 1665-1672.

37. Aiken, R.B. Diel periodicity of song type in an aquatic insect (Palmacorixa buenoi: Heteroptera: Corixidae). *Can. Entomol.* **1985**, *117*, 1569–1572.

38. Aiken, R.B. The Role of Acoustic Signalling in the Aggregating and Mating Behaviour of Palmacorixa Nana Walley (Heteroptera: Corixidae). University of Toronto, 1982.

39. Gogala, M.; Čokl, A.; Drašlar, K.; Blaževič, A. Substrate-borne sound communication in cydnidae (Heteroptera). *J. Comp. Physiol.* **1974**, *94*, 25.

40. Filippi, L.; Hironaka, M.; Tojo, S.; Nomakuchi, S. Insemination success discrepancy between long-term and short-term copulations in the provisioning shield bug, Parastrachia japonensis (Hemiptera: Cydnidae). *J. Ethol.* **2000**, *18*, 29–36.

41. Schwertner, C.F.; Nardi, C. Burrower Bugs (Cydnidae). In *True Bugs (Heteroptera) of the Neotropics. Springer: Boston, USA.*; 2015; pp. 639–680.

42. Hill, L. First record of Dipsocoridae (Hemiptera) from Australia with the description of four new species of Cryptostemma Herrich-Schaeffer. *Aust. J. Entomol.* **1987**, *26*, 129–139.

43. Aldrich, J.R. Chemical Communication in the True Bugs and Parasitoid Exploitation. In *Chemical Ecology of Insects 2. Spinger: Boston, USA.*; 1995; Vol. 2, pp. 318–363.

44. Hickman, V. V.; Hickman, J.L. Observations on the biology ofOncylocotis tasmanicus(Westwood) with descriptions of the immature stages (Hemiptera, Enicocephalidae). *J. Nat. Hist.* **1981**, *15*, 703–715.

45. Polhemus, J.T.; Lindskog, P. The stridulatory mechanism of Nerthra Say, a new species, and synonymy (Heteroptera: Gelastocoridae). *J. New York Entomol. Soc.* **1994**, 242–248.

46. Spence, J.R.; Anderson, N.M. Biology of water striders: interactions between systematics and ecology. *Annu. Rev. Entomol.* **1994**, *39*, 101–128.

47. Moller Andersen, N.M. A phylogenetic analysis of the evolution of sexual dimorphism and mating systems in water striders (Hemiptera: Gerridae). *Biol. J. Linn. Soc.* **1997**, *61*, 345–368.

48. Wilcox, R.S.; Spence, J.R. The mating system of two hybridizing species of water striders (Gerridae). *Behav. Ecol. Sociobiol.* **1986**, *19*, 76–85.

49. Han, C.S.; Jablonski, P.G. Female genitalia concealment promotes intimate male courtship in a water strider. *PLoS One* **2009**, *4*, e5793.

50. Heming-van Battum, K.E.; Heming, B.S. Structure, function, and evolutionary significance of the reproductive system in males ofHebrus ruficeps andH. pusillus (heteroptera, gerromorpha, hebridae). *J. Morphol.* **1989**, *202*, 281–323.

51. Aiken, R.B. Sound Production by Aquatic Insects. *Biol. Rev.* **1985**, *60*, 163–211.

52. Barbosa, J.F.; Rodrigues, H.D.D. The True Water Bugs (Nepomorpha). *Entomol. Focus* **2015**, 159–199.

53. Aldrich, J.R.; Blum, M.S.; Fales, H.M. Species-specific natural products of adult male leaf-footed bugs (Hemiptera: Heteroptera). *J. Chem. Ecol.* **1979**, *5*, 53–61.

54. Austin, A.D.; Yeates, D.K.; Cassis, G.; Fletcher, M.J.; La Salle, J.; Lawrence, J.F.; McQuillan, P.B.; Mound, L.A.; J Bickel, D.; Gullan, P.J.; et al. Insects 'Down Under'–Diversity, endemism and evolution of the Australian insect fauna: examples from select orders. *Aust. J. Entomol.* **2004**, *43*, 216–234.

55. Vázquez, Á. Coreid Bugs and Relatives: Coreidae, Stenocepahidae, Alydidae, Rhopalidae, and Hyocephalidae (Hemiptera: Coreoidea). *Encycl. Entomol.* **2008**, 1058–1062.

56. Schuh, R.T.; Slater, J.A. *True bugs of the world (Hemiptera: Heteroptera): classification and natural history. Cornell University Press: Ithaca, New York, USA.*; 1995;

57. Weirauch, C.; Fernandes, J.A.M. The minute litter bugs (Dipsocoromorpha). In *In True bugs (Heteroptera) of the Neotropics*; 2015; pp. 99–109.

58. Gossner, M.M.; Damken, C. Diversity and ecology of saproxylic Hemiptera. In *Saproxylic Insects*; 2018; pp. 263–317.

59. Schaefer, C.W.; Wilcox, D. Notes on the Morphology, Taxonomy, and Distribution of the Idiostolidae (Hemiptera-Heteroptera). *Ann. Entomol. Soc. Am.* **1969**, *62*, 482–502.

60. Woodward, T.E. The Australian Idiostolidae (Hemiptera: Heteroptera). *Trans. R. Entomol. Soc. London* **2009**, *120*, 253–261.

61. Schaefer, C.W. Some Notes on Heteropteran Trichobothria. *Gt. Lakes Entomol.* **2017**, *1*, 2.

62. Davis, N.T.; Usinger, R.L. The Biology and Relationships of the Joppeicidae (Heteroptera). *Ann. Entomol. Soc. Am.* **1970**, *63*, 577–587.

63. Morimoto, S.; Imamura, T.; Visarathanonth, P.; Miyanoshita, A. Effects of temperature on the development and reproduction of the predatory bug Joppeicus paradoxus Puton (Hemiptera: Joppeicidae) reared on Tribolium confusum eggs. *Biol. Control* **2007**, *40*, 136–141.

64. Oliver, C.; Cordero, C. Multiple mating reduces male survivorship but not ejaculate size in the polygamous insect Stenomacra marginella (Heteroptera: Largidae). *Evol. Ecol.* **2009**, *23*, 417–424.

65. Cuatianquiz, C.; Cordero, C. Experimental manipulation of male behaviour during copulation in Stenomacra marginella (Heteroptera: Largidae): Effect on copula duration, female remating and oviposition. *Behav. Processes* **2006**, *73*, 222–227.

66. Moreno-García, M.; Cordero, C. On the function of male genital claspers in Stenomacra marginella (Heteroptera: Largidae). *J. Ethol.* **2008**, *26*, 255–260, doi:10.1007/s10164-007-0058-8.

67. Schaefer, C.W.; Ahmad, I. Cotton stainers and their relatives (Pyrrhocoroidea: Pyrrhocoridae and Largidae). *Heteroptera Econ. importance* **2000**, 271–308.

68. Eberhard, W.G. Evidence for Widespread Courtship During Copulation in 131 Species of Insects and Spiders, and Implications for Cryptic Female Choice. *Evolution (N. Y).* **1994**, *48*, 711–733.

69. Tatarnic, N.J.; Cassis, G.; Siva-Jothy, M.T. Traumatic Insemination in Terrestrial Arthropods. *Annu. Rev. Entomol.* **2014**, *59*, 245–261.

70. Horton, D.R.; Lewis, T.M.; Dobbs, T.T. Interceptions of Anthocoridae, Lasiochilidae, and Lyctocoridae at the Miami plant inspection station (Hemiptera: Heteroptera). *Florida Entomol.* **2013**, 482–497.

71. Jung, S.H.; Lee, S. New Record of the Family Lasiochilidae Carayon, 1972 (Hemiptera: Cimicoidea) from the Korean Peninsula, with Re-Description of Lasiochilus (Dilasia) japonicusfrom Jeju Island. *J. Asia. Pac. Entomol.* **2007**, *10*, 5–11.

72. McDonald, F.J.D. A new species of Lestoniidae (Hemiptera). *Pacific Insects* **1969**, *11*, 187–190.

73. Fischer, C. The disc-like organ of the Lestoniidae (Heteroptera: Pentatomoidea), with remarks on lestoniid relationships. *Insect Syst. Evol.* **2000**, *31*, 201–208.

74. Cassis, G.; Gross, G.F. *Hemiptera (Vol. 27). CSIRO Publishing: Clayton, Australia.*; 2002;

75. Carpintero, D.L. Minute pirate bugs (Anthocoridae and Lyctocoridae). In *True Bugs (Heteroptera) of the Neotropics. Springer: Dordrecht, The Netherlands.*; 2015; pp. 217–236.

76. Jung, S.; Kim, J.; Lee, H.; Roca-Cusachs, M. Taxonomic review of Lyctocoridae (Hemiptera: Heteroptera: Cimicomorpha) from the Korean Peninsula. *Korean J. Agric. Sci.* **2019**, *46*, 79–84.

77. Panizzi, A.R.; Silva, F.A. Seed-sucking bugs (Heteroptera). In *Insect bioecology and nutrition for integrated pest management. CRC Press: Florida, USA.*; 2012; pp. 295–324.

78. Rodriguez, R.L.; Eberhard, W.G. Male courtship before and during copulation in two species of Xyonysius bugs (Hemiptera, Lygaeidae). *J. Kansas Entomol. Soc.* **1994**, 37–45.

79. Rodriguez, R.L. Possible female choice during copulation in Ozophora baranowskii (Heteroptera: Lygaeidae): female behavior, multiple copulations, and sperm transfer. *J. Insect Behav.* **1998**, *11*, 725–741.

80. Rodriguez, S.; Lucas, R. Copulation, fighting behavior and life cyle of Neopamera bilobata (Heteroptera: Lygaeidae). *Rev. Biol. Trop.* **1998**, *46*, 837–840.

81. McLain, D.K. Host plant density and territorial behavior of the seed bug, Neacoryphus bicrucis (Hemiptera: Lygaeidae). *Behav. Ecol. Sociobiol.* **1984**, *14*, 181–187.

82. Sillén-Tullberg, B. Prolonged copulation: a male 'postcopulatory'strategy in a promiscuous species, Lygaeus equestris (Heteroptera: Lygaeidae). *Behav. Ecol. Sociobiol.* **1981**, *9*, 283–289.

83. Thorpe, K.W.; Harrington, B.J. Sound production and courtship behavior in the seed bug Ligyrocoris diffusus. *Ann. Entomol. Soc. Am.* **1981**, *74*, 369–373.

84. Suzaki, Y.; Miyatake, T. Testing for adaptive explanations of bimodal genital insertion duration in the stalk-eyed seed bug. *Anim. Behav.* **2011**, *82*, 1103–1108.

85. Chopra, N.P.; Rustagi, K.B. The subfamily Chauliopinae of India and Sri Lanka (Hemiptera : Malcidae). *Orient. Insects* **1982**, *16*, 19–28.

86. Yamada, K.; Yamamoto, S.; Takahashi, Y. Aphrastomedes anthocoroides, a remarkable new cimicomorphan genus and species (Hemiptera: Heteroptera) from Upper Cretaceous Burmese amber. *Cretac. Res.* **2018**, *84*, 442–450.

87. Grazia, J.; Simões, F.L.; Panizzi, A.R. Morphology, ontogeny, reproduction, and feeding of true bugs. In *True Bugs (Heteroptera) of the Neotropics. Springer: Boston, USA.*; 2015; pp. 21–55.

88. McDonald, F.J.D. A new species of Megaris and the status of the Megarididae McAtee & Malloch and Canopidae Amyot & Serville (Hemiptera: Pentatomoidea). *J. New York Entomol. Soc.* **1979**, 42–54.

89. Wessel, A.; Mühlethaler, R.; Hartung, V.; Kuštor, V.; Gogala, M. The Tymbal: Evolution of a Complex Vibration-Producing Organ in the Tymbalia (Hemiptera excl. Sternorrhyncha). In *Studying Vibrational Communication. Animal Signals and Communication. Springer: Berlin, Heidelberg, Germany.*; 2014; Vol. 3, pp. 395–444.

90. Wheeler, A.G.; Henry, T.J. Plant Bugs (Hemiptera: Miridae). In *Encyclopedia of Entomology. Springer: Boston, USA.*; 2008.

91. Stork, N.E. The structure and function of the adhesive organs on the antennae of male Harpocera thoracica (Fallen)(Miridae; Hemiptera). *J. Nat. Hist.* **1981**, *15*, 639–644.

92. Aldrich, J.R.; Blum, M.S.; Duffey, S.S.; Fales, H.M. Male specific natural products in the bug, Leptoglossus phyllopus: Chemistry and possible function. *J. Insect Physiol.* **1976**, *22*, 1201–1206.

93. Constantz, G. The Mating Behavior of a Creeping Water Bug, Ambrysus occidentalis (Hemiptera: Naucoridae). *Am. Midl. Nat.* **1974**, *92*, 234–239.

94. Brewer, D.; Sites, R. Behavioral Inventory of Pelocoris femoratus (Hemiptera: Naucoridae). *J. Kansas Entomol. Soc.* **1994**, *67*, 193–198.

95. Keffer, S.L. Systematics of the New World waterscorpion genus Curicta Stål (Heteroptera: Nepidae). *J. New York Entomol. Soc.* **1996**, 117–215.

96. Sites, R.W.; Polhemus, J.T. Nepidae (Hemiptera) of the United States and Canada. *Ann. Entomol. Soc. Am.* **1994**, *87*, 27–42.

97. Choe, J.C.; Crespi, B.J. eds. *The evolution of social behaviour in insects and arachnids. Cambridge University Press: Cambridge, UK.*; 1997;

98. Wilcox, R.S. Sound-producing mechanisms of Buenoa macrotibialis Hungerford (Hemiptera: Notonectidae). *Int. J. Insect Morphol. Embryol.* **1975**, *4*, 168–182.

99. Han, C.S.; Jablonski, P.G.; Brooks, R.C. Intimidating courtship and sex differences in predation risk lead to sex-specific behavioural syndromes. *Anim. Behav.* **2015**, *109*, 177–185.

100. Cheng, L.A.N.N.A.; Frank, J.H. Marine insects and their reproduction. *Oceanogr. Mar. Biol. An Annu. Rev.* **1993**, *31*, 479–506.

101. Schaefer, C.W.; Zheng, L.-Y.; Tachikawa, S. A review of Parastrachia (Hemiptera: Cydnidae: Parastrachiinae). *Orient. Insects* **1991**, *25*, 131–144.

102. Sweet, M.H.; Schaefer, C.W. Parastrachiinae (Hemiptera: Cydnidae) raised to family level. *Ann. Entomol. Soc. Am.* **2002**, *95*, 441–448.

103. Eberhard, W.G. Copulatory courtship and cryptic female choice in insects. *Biol. Rev. Camb. Philos. Soc.* **1991**, *66*, 1–31, doi:10.1111/j.1469-185x.1991.tb01133.x.

104. Lanigan, P.J.; Barrows, E.M. Sexual behavior of Murgantia histrionica (Hemiptera: Pentatomidae). *Psyche A J. Entomol.* **1977**, *84*, 191–197.

105. Zahn, D.K.; Girling, R.D.; McElfresh, J.S.; Cardé, R.T.; Millar, J.G. Biology and reproductive behavior of Murgantia histrionica (Heteroptera: Pentatomidae). *Ann. Entomol. Soc. Am.* **2008**, *101*, 215–228.

106. Capinera, J.L. ed. *Encyclopedia of entomology. Springer: Boston, USA.*; 2008;

107. Schwertner, C.F.; Grazia, J. Less Diverse Pentatomoid Families (Acanthosomatidae, Canopidae, Dinidoridae, Megarididae, Phloeidae, and Tessaratomidae). In *True Bugs (Heteroptera) of the Neotropics. Entomology in Focus, vol 2. Springer: Boston, USA.*; 2015.

108. da Fonseca, F.S.; Salomão, A.T.; Vasconcellos-Neto, J.; Lopes, T.I.; Marsaioli, A.J. Volatile Compounds from the Bark Bugs Phloea subquadrata and Phloeophana longirostris (Heteroptera: Phloeidae). *J. Braz. Chem. Soc.* **2017**, *28*, 1905–1910.

109. Jorigtoo, N.; Schaefer, C.W.; Lockwood, J.A. Stridulatory apparatus of Piesma Le Peletier & Serville (Hemiptera: Piesmatidae). *Ann. Entomol. Soc. Am.* **1998**, *91*, 848–851.

110. Moore, T.E. Audiospectrographic Analysis of Sounds of Hemiptera and Homoptera. *Ann. Entomol. Soc. Am.* **1961**, *54*, 273–291.

111. Drake, C.J.; Davis, N.T. The Morphology and Systematics of the Piesmatidae (Hemiptera), with Keys to World Genera and American Species. *Ann. Entomol. Soc. Am.* **1958**, *51*, 567–581.

112. Hibino, Y. Female choice for male gregariousness in a Stink bug,Megacopta punctissimum (Montandon) (Heteroptera, Plataspidae). *J. Ethol.* **1986**, *4*, 91–95.

113. Hosokawa, T.; Suzuki, N. Mating aggregation and copulatory success by males of the stink bug, Megacopta punctatissima (Heteroptera: Plataspidae). *Appl. Entomol. Zool.* **2000**, *35*, 93–99.

114. Himuro, C.; Hosokawa, T.; Suzuki, N. Alternative mating strategy of small male Megacopta punctatissima (Hemiptera: Plataspidae) in the presence of large intraspecific males. *Ann. Entomol. Soc. Am.* **2006**, *99*, 974–977, doi:10.1603/0013-8746(2006)99[974:AMSOSM]2.0.CO;2.

115. Kovac, D. A Quantitative Analysis of Secretion-Grooming Behaviour in the Water Bug Plea minutissima Leach (Heteroptera, Pleidae): Control by Abiotic Factors. *Ethology* **2010**, *93*, 41–61.

116. Štys, P.; Baňař, P. A new Afrotropical genus of Plokiophilidae with a new free-living species from Madagascar (Hemiptera: Heteroptera). *Entomol. Am.* **2016**, *112*, 220–229.

117. Marshall, A.G. The ecology of the bat ectoparasite Eoctenes spasmae (Hemiptera: Polyctenidae) in Malaysia. *Biotropica* **1982**, 50–55.

118. Polhemus, D.A.; Carrie, R.H. A new species of Potamocoris (Heteroptera: Potamocoridae) from Belize, and synonymy of the genus Coleopterocoris. *Tijdschr. voor Entomol.* **2013**, *156*, 141–149.

119. Jorge, A.S.; Lomônaco, C. Body size, symmetry and courtship behavior of Dysdercus maurus distant (Hemiptera: Pyrrhocoridae). *Neotrop. Entomol.* **2011**, *40*, 305–311.

120. Youdeowei, A. Laboratory studies on the aggregation of feeding Dysdercus intermedius Distant (Heteroptera: Pyrrhocoridae). *Proc. R. Entomol. Soc. London. Ser. A, Gen. Entomol.* **2009**, *41*, 45–50.

121. Zdarek, J. Mating Behaviour in the Bug, Pyrrhocoris Ap Ter Us L.(Heteroptera): Ontogeny and Its Environmental Control. *Behaviour* **1970**, *37*, 253–268.

122. Lima, M.M.; Jurberg, P.; Almeida, J.R.D. Behavior of triatomines (Hemiptera: Reduviidae) vectors of Chagas' disease: I. Courtship and copulation of Panstrongylus megistus (Burm-1835) in the laboratory. *Mem. Inst. Oswaldo Cruz* **1986**, *81*, 1–5.

123. Rojas, J.C.; Malo, E.A.; Gutierrez-Martinez, A.; Ondarza, R.N. Mating Behavior of Triatoma mazzottii Usinger (Hemiptera: Reduviidae) Under Laboratory Conditions. *Ann. Entomol. Soc. Am.* **1990**, *83*, 598–602.

124. Manrique, G.; Lazzari, C.R. Sexual behaviour and stridulation during mating in Triatoma infestans (Hemiptera: Reduviidae). *Mem. Inst. Oswaldo Cruz* **1994**, *89*, 629–633.

125. Vitta, A.C.R.; Lorenzo, M.G. Copulation and Mate Guarding Behavior in Triatoma brasiliensis (Hemiptera: Reduviidae). *J. Med. Entomol.* **2009**, *46*, 789–795.

126. Carroll, S.P. The adaptive significance of mate guarding in the soapberry bug, Jadera haematoloma (Hemiptera: Rhopalidae). *J. Insect Behav.* **1991**, *4*, 509–530.

127. Zych, A.F.; Mankin, R.W.; Gillooly, J.F.; Foreman, E. Stridulation by Jadera haematoloma (Hemiptera: Rhopalidae): Production Mechanism and Associated Behaviors. *Ann. Entomol. Soc. Am.* **2012**, *105*, 118–127.

128. Schwarz, J.; Gries, G. 2-Phenylethanol: Context-specific aggregation or sex-attractant

pheromone of Boisea rubrolineata (Heteroptera: Rhopalidae). *Can. Entomol.* **2010**, *142*, 489–500.

129. Polhemus, J.T. Shore bugs (Hemiptera: Saldi dae, etc.). In *Marine Insects. Cheng, L.*; 1976; pp. 225–262.

130. Scudder, G.G.E. The Schizopteridae (Hemiptera), a family new to Canada. *J. Entomol. Soc. Br. Columbia* **2010**, *107*, 85–86.

131. Numata, H.; Matsui, N.; Hidaka, T. Mating Behavior of the Bean Bug, Riptortus clavatus THUNBERG : Heteroptera : Coreidae : Behavioral Sequence and the Role of Olfaction. *Appl. Entomol. Zool.* **1986**, *21*, 119–125.

132. Spangenberg, R.; Friedemann, K.; Weirauch, C.; Beutel, R.G. The head morphology of the potentially basal heteropteran lineages Enicocephalomorpha and Dipsocoromorpha (Insecta: Hemiptera: Heteroptera). *Arthropod Syst Phyl* **2013**, *71*, 103–136.

133. Schaefer, C.W. The Morphology and Relationships of the Stenocephalidae and Hyocephalidae (Hemiptera: Heteroptera: Coreoidea). *Ann. Entomol. Soc. Am.* **1981**, *74*, 83–95.

134. Myers, J.G. On the systematic position of the family Termitaphididae (Hemiptera, Heteroptera), with a description of a new genus and species from Panama. *Psyche A J. Entomol.* **1924**, *31*, 259–278.

135. Kumar, R. Morphology of the Reproductive and Alimentary Systems of the Aradoidea (Hemiptera), with Comments on Relationships within the Superfamily. *Ann. Entomol. Soc. Am.* **1967**, *60*, 17–25.

136. Dzerefos, C.M.; Witkowski, E.T.F.; Toms, R. Life-history traits of the edible stinkbug, Encosternum delegorguei (Hem., Tessaratomidae), a traditional food in southern Africa. *J. Appl. Entomol.* **2009**, *133*, 749–759.

137. Schaefer, D.B.; Carl, W. A new species of Thaumastellidae (Hemiptera: Pentatomoidea) from southern Africa. *J. Entomol. Soc. South. Afr.* **1971**, *34*, 207–214.

138. Schaefer, C.W. The sound-producing structures of some primitive Pentatomoidea (Hemiptera: Heteroptera). *J. New York Entomol. Soc.* **1980**, 230–235.

139. Schilthuizen, M. The evolution of chirally dimorphic insect genitalia. *Tijdschr. voor Entomol.* **2007**, *150*, 347–354.

140. Wilcken, C.; Soliman, E.; de Sá, L.; Barbosa, L.; Dias, T.R.; Ferreira-Filho, P.; Oliveira, R.R. Bronze bug Thaumastocoris peregrinus Carpintero and Dellapé (Hemiptera: Thaumastocoridae) on Eucalyptus in Brazil and its distribution. *J. Plant Prot. Res.* **2010**, *50*, 201–205.

141. Noack, A.E.; Cassis, G.; Rose, H.A. Systematic revision of Thaumastocoris Kirkaldy (Hemiptera: Heteroptera: Thaumastocoridae). *Zootaxa* **2011**, *3121*, 1–60.

142. Sheeley, R.D.; Yonke, T.R. Biological notes on seven species of Missouri tingids (Hemiptera: Tingidae). *J. Kansas Entomol. Soc.* **1977**, 342–356.

143. Kim, J.; Roca-Cusachs, M.; Jung, S. Taxonomic review of the genus Urostylis (Hemiptera: Heteroptera: Urostylididae) from the Korean Peninsula, with description of a new species. *Zootaxa* **2018**, *4433*, 445.

144. Rider, D.A. Family Urostylididae Dallas, 1851. *Cat. Heteroptera Palaearct. Reg.* **2006**, *5*, 102–116.

145. Van Doesburg, P.H. *A taxonomic revision of the family Velocipedidae Bergroth, 1891 (Insecta: Heteroptera).*; 2004;

146. Kment, P.; Šramek, P. First record of the family Velocipedidae (Heteroptera: Cimicomorpha) from Nepal. *Acta Entomol. Musei Natl. Pragae* **2005**, *45*, 17–18.

147. Panizzi, A.R.; Grazia, J. (Eds). *True Bugs (Heteroptera) of the Neotropics. Vol. 2. Springer: New York, USA.*; 2015;
